# Microbiota in the Rhizosphere and Seed of Rice From China, With Reference to Their Transmission and Biogeography

**DOI:** 10.3389/fmicb.2020.00995

**Published:** 2020-07-10

**Authors:** Xin Zhou, Jin-Ting Wang, Zhi-Feng Zhang, Wei Li, Wen Chen, Lei Cai

**Affiliations:** ^1^State Key Laboratory of Mycology, Institute of Microbiology, Chinese Academy of Sciences, Beijing, China; ^2^College of Life Science, University of Chinese Academy of Sciences, Beijing, China; ^3^College of Marine Life Sciences, Ocean University of China, Qingdao, China; ^4^Ottawa Research and Development Centre, Science and Technology Branch, Agriculture and Agri-Food Canada, Ottawa, ON, Canada

**Keywords:** rice seed, rice rhizosphere, vertical transmission, microbial community, metabarcoding

## Abstract

Seeds play key roles in the acquisition of plant pioneer microbiota, including the transmission of microbes from parent plants to offspring. However, the issues about seed microbial communities are mostly unknown, especially for their potential origins and the factors influencing the structure and composition. In this study, samples of rice seed and rhizosphere were collected from northeast and central-south China in two harvest years and analyzed using a metabarcoding approach targeting 16S rRNA gene region. A higher level of vertical transmission (from parent seed microbiota to offspring) was revealed, as compared to the acquisition from the rhizosphere (25.5 vs 10.7%). The core microbiota of the rice seeds consisted of a smaller proportion of OTUs (3.59%) than that of the rice rhizosphere (7.54%). Among the core microbiota, species in *Arthrobacter*, *Bacillus*, *Blastococcus*, *Curtobacterium*, *Pseudomonas*, and *Ramlibacter* have been reported as potential pathogens and/or beneficial bacteria for plants. Both the seed and the rhizosphere of rice showed distance-decay of similarity in microbial communities. Seed moisture and winter mean annual temperature (WMAT) had significant impacts on seed microbiota, while WMAT, total carbon, available potassium, available phosphorus, aluminum, pH, and total nitrogen significantly determined the rhizosphere microbiota. Multiple functional pathways were found to be enriched in the seed or the rhizosphere microbiota, which, to some extent, explained the potential adaptation of bacterial communities to respective living habitats. The results presented here elucidate the composition and possible sources of rice seed microbiota, which is crucial for the health and productivity management in sustainable agriculture.

## Introduction

Seeds, the most remarkable vectors in plants’ life, enable plants to give rise to new generations (Gundel et al., 2011; Mitter et al., 2017). Seeds have evolved with diverse microorganisms, and the initial microbiome is established during the seed development, maturing, dormancy, and germination periods (Bacilio-Jiménez et al., 2001; Nelson, 2004). The acquisition of microbiota associated with the seed could be through both horizontal and vertical (inheritance) transmissions (Vandenkoornhuyse et al., 2015; Shade et al., 2017), among which the vertical transmission has been shown to have long term impacts on plant growth, development and health (Nelson, 2004; Gundel et al., 2011). For example, Hardoim et al. (2012) found that seed-borne bacteria could improve the rice fitness. Meanwhile, seed may acquire soil- or airborne microorganisms (horizontal transmission) from the surrounding environments, which also play important roles for plant health (Gundel et al., 2011; Shade et al., 2017). For example, when germinating, the seed releases nitrogen-rich compounds, which may attract microbial inhabitants from the surrounding environment in establishing the spermosphere (Nelson, 2004). These attracted microbiota could promote plant germination and growth by producing phytohormone, and/or protect seedlings from soil-borne pathogens at the vulnerable stage (Bacilio-Jiménez et al., 2001). However, the relative importance of horizontal and vertical transmissions that contribute to the rice seed microbiota remains unclear (Shade et al., 2017).

Moreover, seeds are propagules for the next generation plants and a dispersion mean for microbes they carry. A better understanding of the microbial community composition of seeds would be helpful to track the seed-borne pathogens, as well as to recover potentially beneficial microbes for plant growth (Bacilio-Jiménez et al., 2001; Yan et al., 2010; Links et al., 2014; Berg and Raaijmakers, 2018). For example, seed endophytic microorganisms could produce plant growth-promoting hormones (e.g., cytokinins, auxins, and gibberellins), improve a host’s tolerance to abiotic stresses, and dissolve soil micronutrients (e.g., phosphorus, potassium, and zinc) for plant acquisition (Finkel et al., 2017; Walitang et al., 2017; Compant et al., 2019). In addition, *Bacillus* spp. in maize seeds exhibited the ability to produce antifungal compounds and to induce the expression of pathogenesis-related genes in the hosts (Gond et al., 2015). Seeds also carry pathogens that are seed-borne or from their surroundings (Barret et al., 2016; Busby et al., 2017). The inheritance or dispersion of crop pathogens to a new location may result in both yield loss and quality loss. For example, *Xanthomonas oryzae* in rice seeds could sporulate and infect seedlings and nearby healthy seedlings, with blight lesions gradually developed (Lee et al., 2005).

China is the largest rice (*Oryza sativa* L.) producing country. Sustainable domestic production of rice is important for China to achieve food self-sufficiency, which, however, is challenging due to the lack of farmland and outbreaks of diseases. Revealing the composition, function and origins of rice seed microbiota will lead to in-depth understanding of plant-microbe holobiont and how it may affect seed quality, long-term storage, and rice productivity (Nelson et al., 2018). To date, researchers have reported the compositional structure of rhizosphere and phyllosphere microbiota during the vegetative or reproductive stage of rice (Knief et al., 2012; Edwards et al., 2015; Zhang et al., 2019). The microbiota associated with rice seed, however, received little attention, despite its importance for rice health (Shade et al., 2017; Eyre et al., 2019). In addition, few studies have explored the relationships between the seed and rhizosphere microbiota of rice (Compant et al., 2019; Eyre et al., 2019). Although the underlying mechanisms are unclear, the rhizosphere microbiota has shown a resemblance to the seed-associated microbiota in the compositional structure (Nelson et al., 2018). Moreover, recent evidence has confirmed microbial migration between the seed and the rhizosphere, and the two compartments may serve as the microbial repositories for each other (Kong et al., 2019). A comparison between the seed and the rhizosphere microbiota of rice, therefore, would infer the fractional contribution of the two transmission routes to the assembly of indigenous microbiota of rice seeds, and potentially their association with rice development and health.

In this study, we collected seed and rhizosphere soil samples of rice from three major rice-producing provinces in China for two harvest years. Our study aimed to (1) characterize the taxonomic and functional profiles of the microbial communities of rice seed surfaces and rhizosphere soils; (2) to define the core microbiota of the seed and rhizosphere soil samples from different geographical locations, and track the possible transmission paths (horizontal or vertical) of seed microbiota; and (3) to infer the significance of environmental and geographical factors shaping the microbial community.

## Materials and Methods

### Seed and Rhizosphere Sampling

During the 2015 and 2016 growing seasons, we selected five experimental sites situated in three major rice-producing provinces in China, including two in Heilongjiang Province (45°50′24″N, 126°49′12″E, Wuchang; 45°50′24″N, 126°51′7″E, Harbin), two in Hubei Province (30°24′36″N, 114°44′24″E, Wuhan; 30°28′48N, 114°44′24″E, Ezhou), and one in Jiangxi Province (28°46′12″N, 115°49′48″E, Nanchang). The detailed information about rice seed and rhizosphere soil samples included in this study is listed in Supplementary Table S1.

At each experimental site, we collected approximately 1.0 kg of rice seeds, which were placed in a sterile plastic zip-lock bag. The sampled seeds were divided into two batches. One batch was stored at 4°C and used for planting the following year in the same field. The other batch was stored at −80°C in sterile plastic zip-lock bags for DNA extractions. To reveal the vertical transmission of the rice seed microbiota, the seeds collected in 2015 at each site were treated as the parent seeds, and their offspring seeds were harvested in 2016.

The rice roots collected from the fields were placed immediately in sterile zip-lock bags and were transported to the laboratory in iceboxes. The rice roots were vigorously shaken by hand for about 5 min. to remove non-adhering soils, which were collected and used for measuring edaphic properties (soil pH, total carbon, total nitrogen, total phosphorus, available potassium, available phosphorus, Fe_2_O_3_, sodium, aluminum, calcium, and sulfur) using standard protocols^1^. Approximately 1 g of soils within 1 mm of root surface was collected in 100 mL PBS solutions, the precipitate of which was stored in sterile centrifuge tubes at −80°C for further analysis (Edwards et al., 2015). The climate information such as winter mean annual temperature (WMAT) and mean annual precipitation (MAP) were obtained from the China Meteorological Data Service Center (CMDC)^2^. The soil type at each experimental site was obtained from China Soil Database^3^.

### DNA Extraction, Library Construction, and Hiseq Sequencing

A modified supersonic elution protocol from Links et al. (2014) was used to extract the total DNA from the surface of the rice seeds. In brief, 25 g of each sample were submerged in 225 ml of phosphate buffer solution (PBS with 0.5% Triton-X100, Sigma) in a shake flask operated at 150 rpm/min for 1 h, followed by sonication for 45 min. using ultrasonic cleaning equipment (KQ-500DE, Kunshan ultrasonic instrument Co. Ltd.). The liquid fraction was then centrifuged for 40 min. at 4000 g. After the supernatant was removed, the precipitate was used for genomic DNA extraction using the FastDNA^®^ Spin kit following the manufacturer’s instructions (MP Biomedicals, Solon, OH, United States). The genomic DNA was amplified using the primer pair 515F (forward primer: 5′-GTGCCAGCMGCCGCGG-3′) and 806R (reverse primer: 5′-GGACTACHVG GGTWTCTAAT-3′) for targeting bacterial 16S rRNA gene V4 region, followed by two rounds of PCR reactions. In the first round PCR, the genomic DNA was amplified using the Phusion High-Fidelity PCR Master Mix (New England Biolabs) with 0.2 μM of forward and reverse primers and 10 ng of template DNA. The reaction conditions consisted of an initial denaturation of 95° for 2 min., followed by 25 cycles of 95°C for 30 s, 55°C for 30 s, and 72°C for 60 s, a final extension at 72°C for 10 min., and held at 4°C. The first-round PCR was performed in triplicates for each sample to reduce PCR biases. The second-round PCR was performed using NEB Next Ultra DNA Library Prep Kit for Illumina (New England Biolabs, United States) following the manufacturer’s recommendations to add index sequences. All PCR products were purified using the GeneJET Gel Extraction Kit (Thermo Fisher Scientific, United States) before sequencing. The library quality was assessed using a Qubit@ 2.0 Fluorometer (Life Technologies, CA, United States) and Agilent Bioanalyzer 2100 system (Annoroad Gene Technology Corporation). Illumina HiSeq sequencing (Illumina Inc., San Diego, CA, United States) was carried out at Annoroad Gene Technology Corporation (Beijing, China), which generated paired-end 2 × 250 bp raw sequencing reads.

### Sequencing Data Processing

The paired-end raw sequencing reads were processed using USEARCH10 software and QIIME 1.9.1 platform (Caporaso et al., 2010; Edgar, 2013). All paired-end sequences were quality filtered using *fastq_filter* and joined by *fastq_mergepairs* scripts. All the chimeric sequences were removed using UCHIME against RDP (Ribosomal Database Project) Gold database (Edgar, 2013). The non-chimeric sequences were sorted by abundance, dereplicated and clustered to the operational taxonomic units (OTUs) using the UPARSE algorithm at 97% sequence similarity. OTUs with <8 reads were removed to minimize PCR or sequencing errors (Edgar, 2013). The representative sequences of the OTUs were assigned to taxonomic lineages by comparing with the SILVA 16S rRNA database (132 release) using the *sintax* command in USEARCH (Edgar, 2013) at the confidence threshold of 0.8. For functional prediction using PICRUSt (v1.1.4, Langille et al., 2013), a new OTUs table was constructed using the *pick_closed_reference_outs* function in QIIME 1.9.1 against the Greengenes database (GG13.5) (Caporaso et al., 2010). The OTU abundance table was first normalized by 16S rRNA gene copy numbers and was used for functional annotation using the *predict_metagenones.py* command.

### Statistical Analysis

Most statistical analysis was carried out using the vegan package (Oksanen et al., 2016) in the R environment (version 3.5.1) (R Core Team, 2017) unless stated otherwise. The rarefaction curves were generated using the *rarecurve* function. The OTU table was rarefied to the minimum reads of samples (*n* = 44,448) using the *rrarefy* function, then the alpha-diversity indices were calculated. The beta-diversity was estimated using the weighted and unweighted UniFrac distances (Lozupone and Knight, 2005) and visualized with the principal coordinates analysis (PCoA) plots. Differences in community composition between the different groups were measured by the ANOSIM (analysis of similarities) and PERMANOVA (multivariate permutational analysis of variance) using the *anosim* and *adonis* functions. The distance-decay model of microbiota was performed using the *betapart* package (Baselga and Orme, 2012). The relative importance of geographic location, climatic condition, and soil physicochemical properties were measured by variation-partitioning analysis using the *varpart* function in *vegan*. The relationship between the Bray-Curtis dissimilarity of samples and the log(x + 1) transformed environmental variables were evaluated by distance-based redundancy analysis (db-RDA) using the *capscale* function (Legendre and Gallagher, 2001) in vegan. The *vif.cca* function was used to resolve the collinearity of all environmental variables, the variables with VIF value below 10 were chosen to build the final model (Oksanen et al., 2016). The statistical significance (alpha = 0.05) of the final model and the terms (i.e., environmental variables) was tested using ANOVA (999 random permutations).

To identify and visualize the OTUs enriched specifically in rice seeds or rice rhizosphere soils at different geographic locations, the ternary plots were generated with the mean values of relative abundance (>0.2% threshold, transformed by log2) using the *limma* package in R, as previously described (Bulgarelli et al., 2015). The edgeR package (Robinson et al., 2010) was used to identify significant changes in KEGG pathways between the rice seed and rice rhizosphere soil samples. The PICRUSt counts table was fitted with a negative binomial generalized log-linear model, and the differential abundance of each functional pathway was tested using the glmFit function. The false discovery rate (FDR) was controlled by adjusting the *p*-values using the Benjamini-Hochberg method. The FEAST (fast expectation-maximization for microbial source tracking) software was used to track the possible sources that contributed to the rice seed microbiota with the default setting (Shenhav et al., 2019). All plots and graphs were generated using the ggplot2 package (Wickham, 2016). The core microbiome was defined as a set of bacterial taxa shared by more than 90% samples in a given treatment group (Eyre et al., 2019).

### Data Accessibility

All raw paired-end Illumina HiSeq sequences are available through the Genome Sequence Archive (GSA) data repository^4^ under the BioProject ID: PRJCA002172 and the GSA accession ID CRA002311.

## Results

### The Overall Richness and Compositional Structure

**FIGURE 1:**
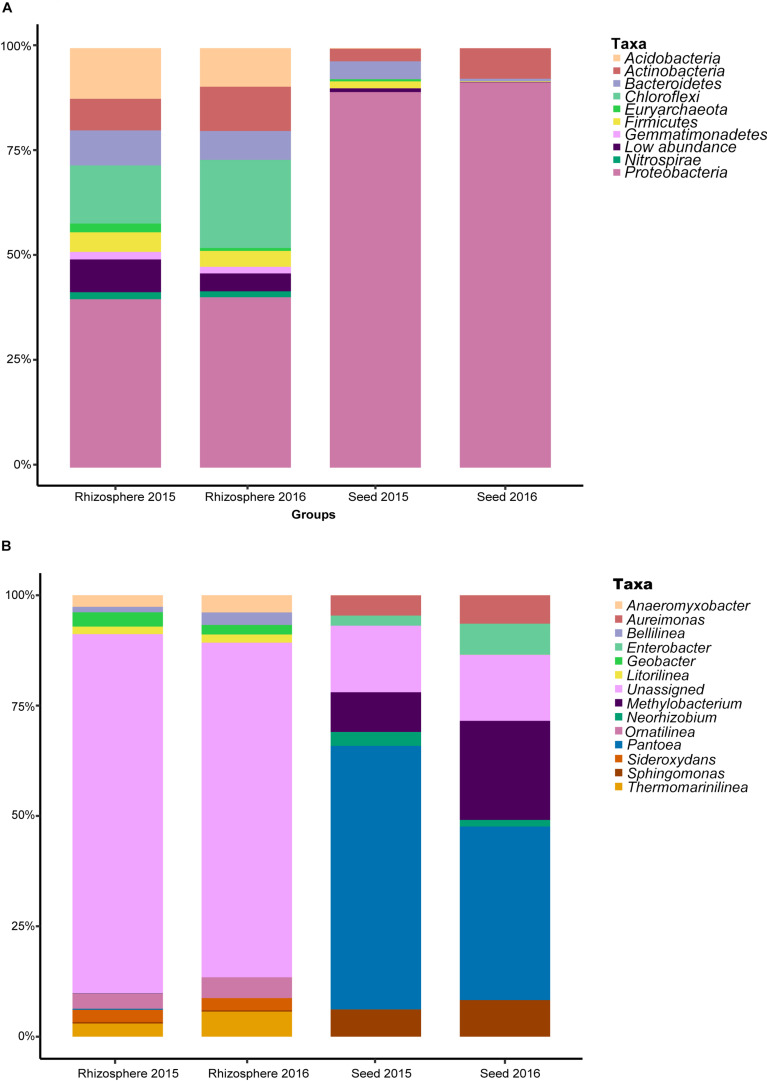
The relative abundance of the most predominant bacterial taxa at the **(A)** phylum, and **(B)** genus levels.

After sequence quality control, a total of 6,715,879 metabarcodes were retained with an average of 61,993 sequences per sample. All non-chimeric sequences were clustered into 10,514 OTUs. After the removal of OTUs with <8 reads, 7,806 OTUs were retained with an average of 50,478 sequences per sample (range: 44,448–69,843). The rarefaction curves of most samples nearly reached the asymptotes, indicating sequencing sufficiency for revealing the true diversity of the samples (Supplementary Figure S1). All the OTUs were assigned to Achaea or Bacteria. For the seed microbiota, the phyla Proteobacteria (90.71% of the total sequences), Actinobacteria (5.14%) and Bacteroidetes (2.44%) were the most predominant (Figure 1A). For the rhizosphere microbiota, Proteobacteria (40.40%), Chloroflexi (17.44%), Acidobacteria (10.62%), Actinobacteria (9.04%), Bacteroidetes (7.61%), and Firmicutes (4.22%) were the most abundant. Interestingly, approximately 63.52 and 12.91% sequences could not be assigned to the genus level for the rhizosphere and seed samples, respectively (Figure 1B), indicating many more unclassified taxa in the rhizosphere.

### Composition and Transmission of Rice Microbial Community

**FIGURE 2:**
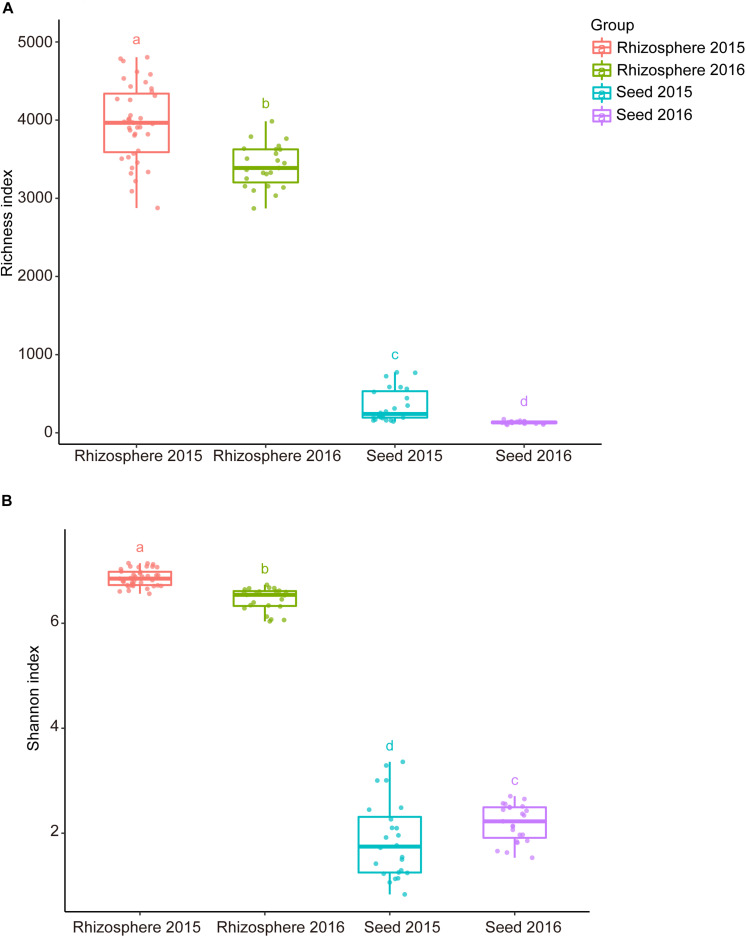
The alpha diversity indices of the seed and rhizosphere microbial communities. **(A)** Richness (number of OTUs); **(B)** Shannon-Wiener index. The lines inside boxes represent the median. The top and bottom whiskers indicate the 5th and 95th percentiles. Means were compared by one-way ANOVA, Tukey’s HSD and LSD-t comparisons. Different lowercase letters on top of the boxplots designate bacterial communities differed significantly in alpha-diversity (alpha = 0.05).

**FIGURE 3:**
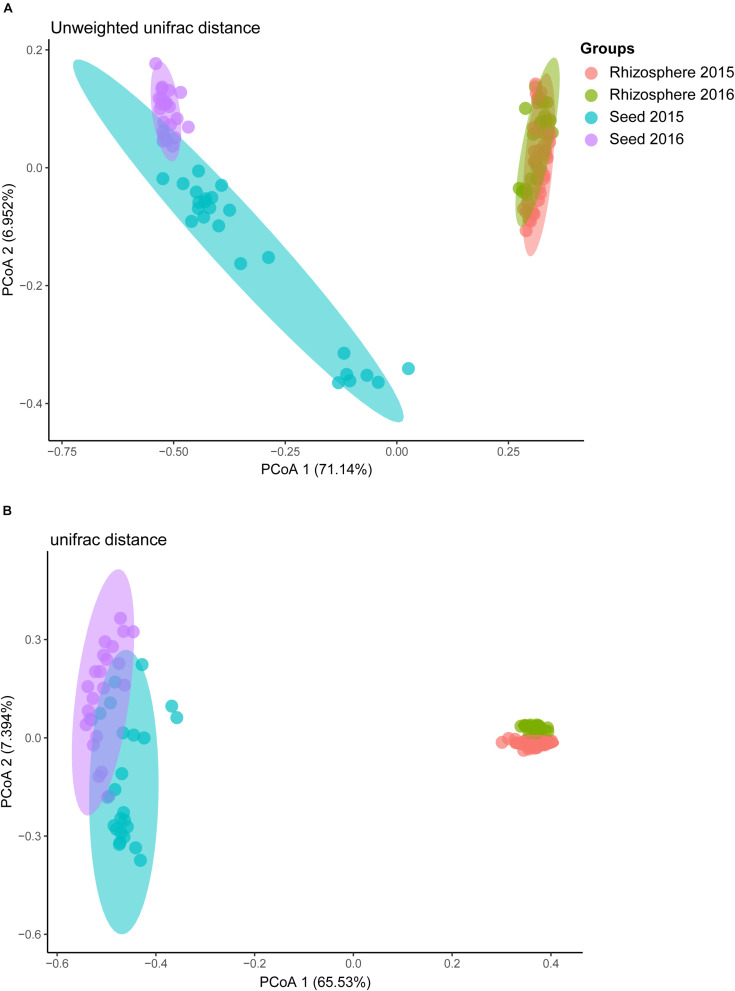
The principal co-ordinate analysis (PCoA) of rice bacterial communities based on **(A)** unweighted, and **(B)** weighted UniFrac distance dissimilarity.

Comparative analyses of the alpha- and beta-diversity were performed using the rarefied OTU table with even sequencing depth (*n* = 44,448). The Shannon index and OTU richness of the rhizosphere microbiota were significantly higher than those of the seed microbiota (Tukey HSD test, *P* < 0.05) (Figures 2A,B). Based on unweighted and weighted UniFrac distance, the PCoA analyses clustered the seed and rhizosphere samples in to two distinct groups (Figures 3A,B), which was further supported by both the PERMANOVA (adonis *P* < 0.001) and the ANOSIM (*r* = 0.7635, *P* = 0.001) analyses. Additionally, the ANOSIM tests showed that the seed microbiota compositional structure differed significantly among samples collected from different geographical locations (*r* = 0.7635, *P* = 0.001). No significant difference in community composition was observed between seeds collected from different years (*r* = −0.003, *P* = 0.429). Such observation indicates that the community compositions were more similar between the parent and the offspring seeds than between the seeds collected from different locations. Using the seed and rhizosphere microbiota in 2015 as the possible sources, the FEAST analysis revealed that the former had higher contribution (25.5% in average, from 10.1 to 34.2%) to the seed microbiota in 2016 than the latter did (10.7% in average, from 5.6 to 17.8%) (Supplementary Table S2), suggesting that the microbiota of the parent seeds is the main source of the offspring seed microbiota.

### Geographic and Environmental Factors Strongly Influenced Microbial Communities

**FIGURE 4:**
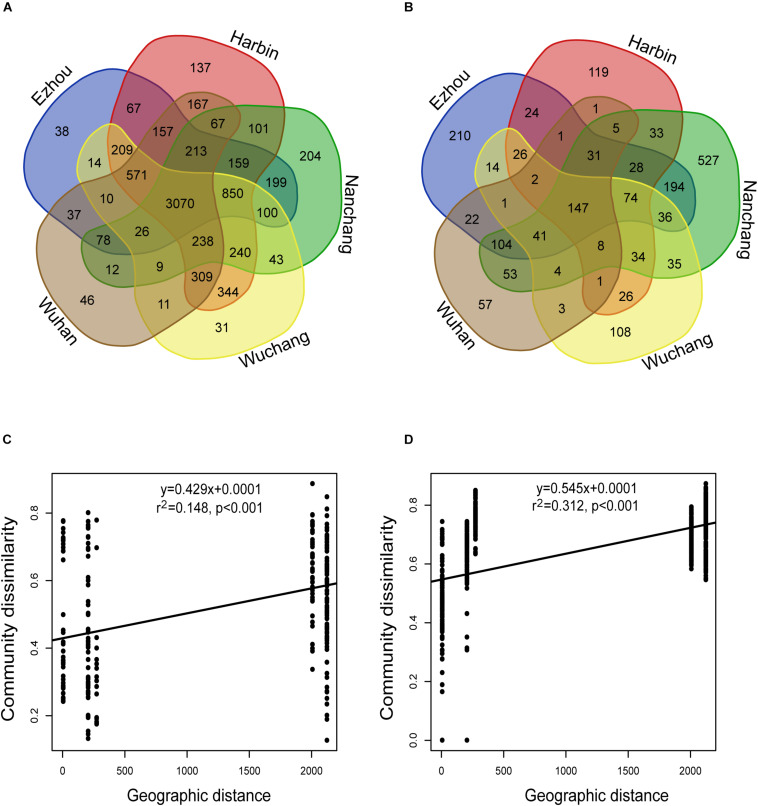
The impact of geographic location on the seed and rhizosphere bacterial communities. **(A,B)** The Venn diagram showing the bacterial OTUs recovered in **(A)** seeds, and **(B)** rhizosphere soils collected from different experimental sites. **(C,D)** The distance-decay relationships based on the Bray-Curtis distance-based community dissimilarities of the **(C)** seed samples: *y* = 0.4293x + 0.0001; *r*^2^ = 0.148; *P* < 0.001; and **(D)** rhizosphere soil samples: *y* = 0.545x + 0.0001; *r*^2^ = 0.312; *P* < 0.001.

For the rhizosphere microbiota, about 39.7% of the total OTUs were recovered from all locations, and about 1.2% (ranged from 0.4 to 2.6%) on average were geographically specific (Figure 4A). For the seed microbiota, however, only 12.4% of the total OTUs were found at all the locations, and about 4.7% on average (ranged from 0.95 to 12.95%) were geographically specific (Figure 4B). In addition, the community variation of the rhizosphere microbiota (*r*^2^ = 0.312, *P* < 0.001) showed a steeper distance-decay relationship (higher divergence in species composition through space) than that of the seed microbiota (*r*^2^ = 0.148, *P* < 0.001) (Figures 4C,D). The environmental factors selected to build the db-RDA models and their influences on community composition are summarized in Supplementary Table S3. For the seed microbiota, WMAT and seed moisture were the primary determinants, accounting for 32.61% of the total community variation. For the rhizosphere microbiota, WMAT, pH, total nitrogen, total carbon, available potassium, available phosphorus and aluminum, as ranked by importance, showed significant influences (*P* < 0.05), accounting for 41.77% of the total variation (Supplementary Table S3).

### Defining and Characterizing the Core Microbiota of Seed and Rhizosphere

**FIGURE 5:**
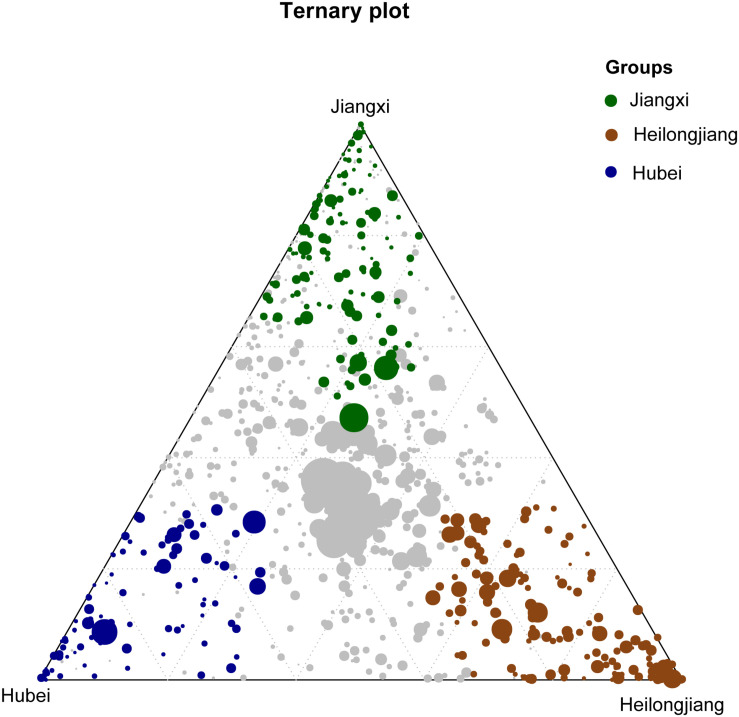
Ternary plot showing bacterial OTUs significantly enriched in Heilongjiang Province (brown filled circles), Hubei Province (blue filled circles) and Jiangxi Province (green filled circles) in the rice rhizosphere soil samples. The gray dots in the center of the ternary plot represent the non-significant OTUs shared by all samples.

The OTUs shared by 90% of all seed or rhizosphere soil samples were selected as the core microbiota (Eyre et al., 2019). The seed core microbiota consisted of 3.59% (38/1058) of the total OTUs which represents 26 bacterial genera, while the rhizosphere core microbiota consisted of 7.54% of total OTUs (583/7733) which represents 253 genera (Supplementary Table S4). Of the core microbiota, the most abundant bacterial genera associated with the rice seed (relative abundance >0.5%) were *Coprobacillus*, *Curtobacterium*, *Kineococcus*, *Pantoea*, *Pseudomonas*, *Quadrisphaera*, *Streptophyta*, *Methylobacterium*, *Neorhizobium*, *Ramlibacter*, *Sphingomonas*, and *Xanthomonas* (Supplementary Table S4). The most abundant (>0.5%) bacterial genera associated with the core microbiota of the rice rhizosphere were *Anaeromyxobacter*, *Arenimonas*, *Arthrobacter*, *Bacillus*, *Bellilinea*, *Blastococcus*, *Bradyrhizobium*, *Curtobacterium*, *Gaiella*, *Nitrospira*, *Noviherbaspirillum*, *Pantoea*, *Paraherbaspirillum*, *Pelagibius*, *Povalibacter*, *Pseudomonas*, *Ramlibacter*, *Sphingosinicella*, *Streptophyta*, and *Thiobacillus* (Supplementary Table S4). We further identified 163 OTUs, 93 OTUs, and 128 OTUs that were significantly enriched (*P* < 0.05) in Jiangxi, Hubei, and Heilongjiang provinces, respectively (Figure 5). The relative abundances of these OTUs in different samples are listed in Supplementary Table S5. Compared with the other provinces, more OTUs enriched in the samples from Heilongjiang Province (northeast China) were found to affiliate with known plant growth–promoting bacteria and/or biocontrol agents, e.g., *Arthrobacter* spp. and *Thermomonas* spp.

### Functional Prediction

**FIGURE 6:**
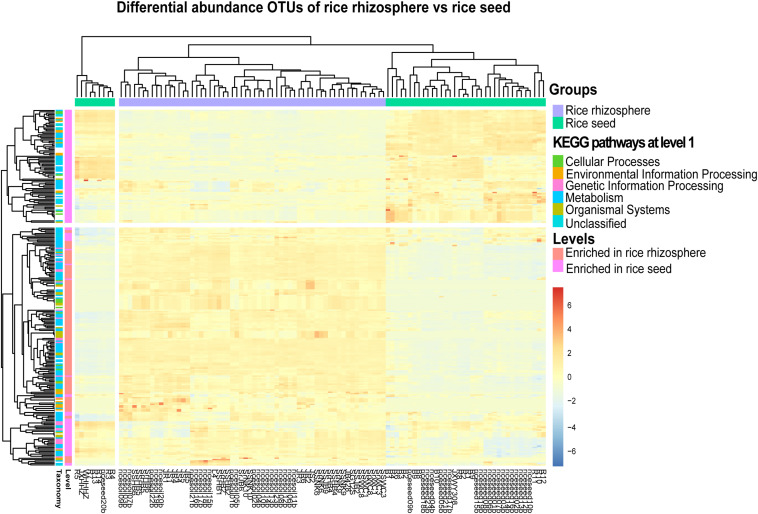
The heatmap representing the PICRUSt-predicted bacterial functional groups differed significantly (FDR < 0.05) in abundance between the rice seed and rhizosphere soil microbiota, based on moderated estimation of log2-fold changes of the CPM-normalized read counts. The higher the abundance of a level-1 KO group in a sample, the deeper the color in the heatmap.

In total, 329 level-3 KEGG Orthology (KO) groups were predicted for both the seed and rhizosphere microbiota. The most abundant KEGG pathways and functions across all samples were affiliated to transporters, ABC transporters, two-component system, DNA repair, recombination proteins, bacterial motility proteins, secretion system, and purine metabolism. The heatmap showed that the rhizosphere and seed samples formed distinct clusters based on their functional profiles and enriched in different metabolism pathways (Figure 6). For example, 131 specific level-3 KO groups were significantly enriched in the rhizosphere soil samples, while 130 were significantly enriched in seed samples (Figure 6 and Supplementary Table S6). Among the significantly enriched level-2 KO groups, those functionally associated with cell communication, excretory system, sensory system, transcription, transport, and catabolism were only predicted from the rhizosphere microbiota, while those associated with membrane transport, and nucleotide metabolism were only predicted from the rice seed microbiota (Supplementary Table S6). Furthermore, the rhizosphere microbiota appeared to be enriched in pathways involved in carbon fixation, energy metabolism, tryptophan biosynthesis, and amino sugar and nucleotide sugar metabolism. Several metabolism pathways involved in the degradation of pesticides and/or chemical fertilizers, e.g., aminobenzoate, butanoate, benzoate, and propanoate, were also found to be enriched in the rhizosphere microbiota. By contrast, the rice seed microbiota was enriched in pathways affiliated to membrane transport (ABC transporter-dependent pathways, ion coupled transporter pathways), the signaling and cellular processes (bacterial motility proteins, two component system, secretion system), genetic information processing (DNA repair and recombination proteins, ribosome biogenesis), or amino acid metabolism (purine metabolism, arginine and proline metabolism, pyrimidine metabolism, peptidases, and amino acid–related enzymes) (Supplementary Table S6).

## Discussion

In this study, we characterized the microbial communities associated with rice seeds and rice rhizosphere soils in three major rice-producing provinces in China for two consecutive years. Studies on seed microbiota were relatively scarce, compared with those on root-associated microbial communities (Edwards et al., 2015; Eyre et al., 2019; Zhang et al., 2019). Previous studies have revealed that microbes could be stably integrated into seeds and transmitted to the offspring generations (Hardoim et al., 2012; Mitter et al., 2017). A better understanding of seed microbiota and their possible major sources (parent seed, air, rain, or rhizosphere) may serve as an important knowledge base for plant health, quality, and disease management and control (Compant et al., 2019). However, seed microbiota is highly variable in both space and time (Guo et al., 1998; Nelson, 2004; Shade et al., 2017). In natural ecosystems, the most important sources, which contributed to seed acquisition of microbes were mediated by soil, animals, wind, and rainfall via horizontal transmission (Klaedtke et al., 2016; Nelson et al., 2018; Compant et al., 2019). By contrast, in an agricultural ecosystem with homogenized landscape and biodiversity evenness, the FEAST analysis suggested that about 25.5% of the seed microbiota was likely inherited from the parent seed (first-year samples) (vertical transmission) and about 10.7% of the seed microbiota was likely acquired from the rhizosphere soil microbiota (horizontal transmission). Our results suggest that the vertical transmission contributed more than horizontal transmission to the assembly of seed microbiota of cultivated rice. Considering the rice rhizosphere compartment is underwater, the rhizosphere microbiota is mainly composed of anaerobic or facultative anaerobic microbes that may not survive on the surface of the seeds (Edwards et al., 2015). We noted that more than 60% of the seed microbiota could not be tracked for their sources, which may warrant other potential acquisitions of the seed microbiota from an environment, e.g., microbes carried and distributed by winds, rains, and animals (Nelson, 2004; Shade et al., 2017; Nelson et al., 2018).

Some seed-transmitted microorganisms have shown beneficial effects on rice plants (Eyre et al., 2019). Pre-treatment of seeds with beneficial microbial inoculants to increase resistance to pathogens and to promote plant growth has been used to enhance seed and plant performance (Finkel et al., 2017; Berg and Raaijmakers, 2018; Zvinavashe et al., 2019). Previous studies have reported that *Flavobacterium* spp. and *Methylobacterium* spp. from rice seeds promoted nitrogen-fixing and methane oxidation, and consequently promoted rice plant growth (Tani et al., 2015). Certain seed-transmitted *Arthrobacter* spp. have been documented for their antifungal activities against the destructive rice pathogens *Rhizoctonia solani* and *Pyricularia grisea* (Cottyn et al., 2009). Our study recovered several core taxa of rice seeds belonging to *Arthrobacter*, *Bacillus, Blastococcus*, *Curtobacterium*, *Pseudomonas*, and *Ramlibacter*, isolates of which have shown *in vitro* and/or *in planta* beneficial effects on promoting the growth, productivity and/or disease resistance of plants (Finkel et al., 2017; Mitter et al., 2017; Pascale et al., 2020). In addition, both the seeds and the rhizosphere soils at different geographic locations harbored rather distinct microbial communities. In particular, we found that the seeds from Northeast China (Heilongjiang Province) were enriched in potentially beneficial *Arthrobacter* spp., *Flavobacterium* spp. and *Methylobacterium* spp., which may be inherited from mother plants or acquired from the environment. Although speculative, the existence of these enriched microorganisms may be associated with the high quality of the rice seeds and the most fertile soils in Northeast China.

On the other hand, seed transmission serves as one of the major dispersal mechanisms for plant pathogens, which could transiently colonize the seed (seed-borne) or be transmitted to the seedling (Darrasse et al., 2007). Torres-Cortés et al. (2019) reported the transmission of two plant pathogens, a fungus *Alternaria brassicicola* and a bacterium *Xanthomonas campestris*, from the radish seeds to the plantlets. In the present study, we found that several potential rice pathogens, such as *Pantoea* spp. and *Xanthomonas* spp., could be carried by parent seeds and inherited by offspring. Since the seed-borne phytopathogens may be transmitted to the seedling and to offspring through vertical transmission, the emergence and the spread of the disease could be in a persistent manner and hard to be eradicated (Elmer, 2001; Barret et al., 2016). Environmental conditions have been considered as critical elements in determining the microbial community associated with rice microbiota (Edwards et al., 2015; Shade et al., 2017). Our results showed that the seed and rhizosphere microbiota were determined by a different set of environmental factors, except for the winter mean annual temperature (WMAT), which was recognized as the primary determinant of both the rhizosphere and the seed microbiota microbiota. Previous studies have shown that the annual average temperature could affect the distribution of *Fusarium graminearum*, one of the major causal agents of *Fusarium* Head Blight (FHB) of wheat. FHB epidemics occur more frequently in the warmer regions of China (Qu et al., 2008). The extremely low winter temperature in Northeast China (−15°C) may impede overwintering survival of plant pathogens in soils and plant residues and provide a healthy environment for the growth of rice plants.

The aboveground seed compartment and the belowground (sometimes below water for rice plants) root rhizosphere soil compartment represent two extremely different environmental habitats (Pascale et al., 2020). Microorganisms associated with different compartments have evolved to develop specific functional traits essential for the adaptation and survival in respective ecological niches (Vandenkoornhuyse et al., 2015; Shade et al., 2017). In contrast to the rhizosphere soil habitats, the seed compartments are characterized by different yet harsher conditions such as desiccation, exposure to UV radiation and oligotrophy (Vandenkoornhuyse et al., 2015). The accumulation of chemical fertilizers, heavy metals, and pesticides in fields may cause the deterioration of farmlands, the pollution of water bodies, and the enormous damage to plant health and productivity (Kuiper et al., 2004). Previous studies have reported the enrichment of microbial pathways or functional groups associated with the phytoremediation capacity of the plants (Fan et al., 2018; Pascale et al., 2020). Similarly, our study revealed the enrichment of multiple energy metabolism pathways in the rhizosphere microbiota, which could potentially promote the absorption and utilization of carbon sources, the acquisition of nitrogen, or the degradation of chemical pollutants (e.g., aminobenzoate, butanoate, benzoate, and propanoate), as also suggested by Finkel et al. (2017) and Pascale et al. (2020). In addition, root associated microbes may promote plant growth by modulating the production of hormone/hormone-like molecules (Pascale et al., 2020). For example, the rhizosphere microbiota enriched in tryptophan biosynthesis pathway may suggest the production of Indole-3-acetic acid (IAA), which could facilitate plant growth and production (Islam et al., 2016). By contrast, seed microbiota was enriched in pathways that may help the seeds adapt to harsh environments (Giuliani et al., 2011). For example, the potentially upregulated pathways involved in DNA repair, the two-component system, secretion system, and ribosome biogenesis, could enable or improve the stress tolerance of seeds under long exposure to the solar UV radiation and the colonization of alien microorganisms on the rice plant (Jacobs and Sundin, 2001).

## Conclusion

The present study has largely improved our understanding of rice seed–associated microbiota and their potential transmission, which forms the foundation for research on developing ecofriendly measures for the control of seed-transmitted phytopathogens or for the enhancement of seed quality and plant performance of rice. This study showed that vertical transmission from parent seeds contributed more to the assembly of rice seed microbiota relevant to the horizontal acquisition from the rhizosphere soils. This study also recovered some potentially beneficial microbes in rice seeds, especially in those from the Northeast region. Potentially enriched metabolism pathways in the seed and rhizosphere microbiota may be important adaptive evolutionary traits of respective bacterial communities. Beyond the scope of this study, the taxonomic identity and biological characteristics of the functional guilds (e.g., *Bacillus* spp., *Pseudomonas* spp.) could be accurately defined using culture-based approaches; following which, the potential roles of the isolates in pathogen transmission and suppression, disease resistance, and/or plant growth-promotion can be assessed using *in vitro* and *in planta* tests as described elsewhere (Mitter et al., 2017; Torres-Cortés et al., 2019; Zhang et al., 2019).

## Data Availability Statement

All the 16S raw data has been deposited into BIG Sub (https://bigd.big.ac.cn/gsub/) under accession number PRJCA002172.

## Author Contributions

LC and WC planned and designed this study. XZ carried out the field and wet-lab experiments with the assistance of Z-FZ, and J-TW. XZ performed the data analysis under the supervision of WC and drafted the manuscript. WL contributed to the data analysis. WC and XZ revised the manuscript. All co-authors read and approved the final manuscript.

## Conflict of Interest

The authors declare that the research was conducted in the absence of any commercial or financial relationships that could be construed as a potential conflict of interest.

## Footnotes

1 http://vdb3.soil.csdb.cn/

2 http://data.cma.cn/en

3 http://vdb3.soil.csdb.cn/

4 http://gsa.big.ac.cn/

## Supplementary Material

FIGURE S1 The rarefaction curves of each sample.

TABLE S1 The metadata associated with rice seed and rhizosphere samples.

TABLE S2 The contributions of rice rhizosphere and parent seeds to the microbiota of offspring based on FEAST analysis.

TABLE S3 The db-RDA models built to assess the effects of environmental variables on rice seed and rhizosphere microbiota. -: the respective variable was not selected for the model; ^∗^*P* < 0.05; ^∗∗^*P* < 0.01; ^∗∗∗^*P* < 0.001.

TABLE S4 The core microbiota of rice seed and rhizosphere samples. The core microbiota is defined as OTUs recovered in 90% samples in a given treatment group.

TABLE S5 The OTUs significantly enriched at different experimental sites in the three provinces.

TABLE S6 The KEGG pathways significantly enriched in rice seed and rhizosphere soil microbiota based on PICRUSt prediction.
