# Supplementary figures and images for: Microbiota in the Rhizosphere and Seed of Rice From China, With Reference to Their Transmission and Biogeography
Source: Front Microbiol. 2020 Jul 10;11:995. doi: 10.3389/fmicb.2020.00995 (PMC7365946; doi:10.3389/fmicb.2020.00995)

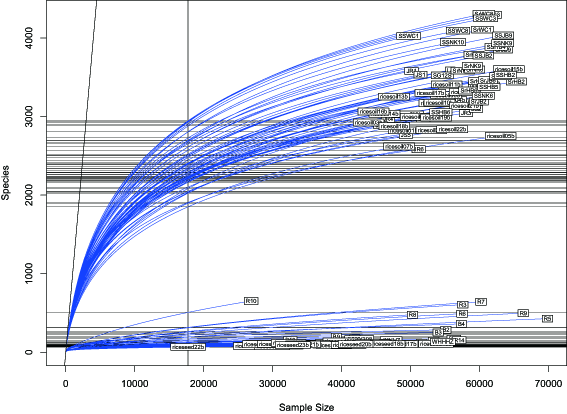

Supplement: FIGURE S1 — The rarefaction curves of each sample. [file Image_1.tif]
